# The effect of significant weight loss after bariatric surgery on echocardiographic indices: an observational study focusing on left ventricular deformation by 2D speckle echocardiography and right ventricular size

**DOI:** 10.1186/s43044-024-00474-6

**Published:** 2024-04-08

**Authors:** Saeed Safari, Mozhgan Parsaee, Mohammad Moradi, Mahdi Hakiminejad, Parisa Koohsari, Farnoosh Larti

**Affiliations:** 1 General Surgery Department, Firoozgar Hospital, Iran University of Medical Sciences, Tehran, Iran; 2 Rajaie Cardiovascular Medical and Research Center, Iran University of Medical Sciences, Tehran, Iran; 3 Cardiology Department, Imam Khomeini Hospital Complex, Tehran University of Medical Sciences, End of Keshavarz Boulevard, Tehran, 1419733141 Iran

**Keywords:** Weight Loss, Bariatric Surgery, 2D Speckle Echocardiography, Left ventricle, Right Ventricular Size, GLS

## Abstract

**Background:**

Obesity is a known risk factor for atherosclerosis and cardiac disease.

**Hypothesis:**

This study evaluated the effect of significant weight loss following bariatric surgery on myocardial deformation indices and right ventricular size (RV). This was a prospective cohort study. Morbid obese patients scheduled for bariatric surgery from July 2017 to February 2018 at Firoozgar Hospital were included in our study and referred for transthoracic echocardiography at Rajaie Cardiovascular Medical and Research Center.

**Results:**

Thirty-four patients entered the study. The absolute value of global longitudinal strain (GLS) at baseline, 3, and 6 months after surgery was 17.42 ± 2.94%, 18.24 ± 3.09%, and 19.52 ± 2.78%, respectively, with a statistically significant difference from baseline to after six months (*P* value < 0.001). The absolute value of global circumferential strain (GCS) at baseline, 3, and 6 months after surgery was 20.14 ± 4.22%, 23.32 ± 4.66%, and 24.53 ± 4.52%, respectively, with statistically significant changes (*P* value < 0.001) from baseline to three months and from baseline to six months and no significant difference from three months to six months. A significant decrease was reported in mechanical dispersion of circumferential strain (38.05 ± 23.81–23.37 ± 20.86 ms, *P* value = 0.006) 6 months after surgery. Right ventricular size three- and six-month post-surgery showed a significant decrease relative to baseline echocardiography.

**Conclusions:**

Bariatric surgery could enhance cardiac function, as proven by 2D speckle echocardiography. Changes in RV size may be related to weight loss and should be considered when assessing patients who have undergone bariatric surgery.

## Background

Obesity is recognized as a risk factor for cardiac dysfunction, atherosclerosis, cardiovascular disease, dyslipidemia, and diabetes mellitus. LV (left ventricular) dilation, LVH (left ventricular hypertrophy), HF (heart failure), and systolic and diastolic dysfunctions are significant obesity-induced changes [1, 2]. Early detection of these cardiovascular abnormalities is important because appropriate treatment may reverse the process. If untreated, it can increase the mortality rate [3].

In the last decade, obesity prevalence has increased considerably worldwide, and medical treatments and lifestyle changes could not completely solve the problem [4]. Therefore, bariatric surgery has been widely accepted, and in 2018, 252,000 bariatric surgeries were performed in the USA [5].

There is a clear association between obesity, arterial hypertension, and left ventricular hypertrophy. It has been reported that systemic hypertension is more detected in obese individuals than those with normal weight [6, 7]. With increasing left ventricular preload, the Frank-Starling curve shifts to the left, leading to cardiac dilation. This leads to left ventricular wall stress as well. The myocardial mass would increase, leading to hypertrophy [8–10]. A well-conducted meta-analysis by Cuspidi in 2014 showed a decrease in LV mass and relative wall thickness, an improvement of LV diastolic function, as reflected by an increase in mitral E/A ratio, a reduction of left atrium diameter, and no changes in LV ejection fraction with weight loss [11].

**Fig. 1:**
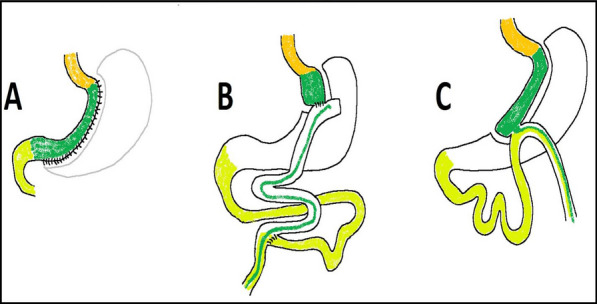
The most common bariatric surgeries are: **A** “Sleeve gastrectomy” consists of creating a tubular stomach and resecting the remaining part. **B** “Roux-en-Y gastric bypass” or RYGB consists of creating a 40-cc small gastric pouch and Roux-en-Y anastomosis of the pouch to the jejunum, 100 cm after Treitz’s ligament. **C** “One anastomosis gastric bypass” or OAGB (formerly, mini-gastric bypass or MGB) consists of the creation of a long and narrow gastric pouch (250 cc) with loop anastomosis to the jejunum, 150 cm after Treitz’s ligament

As speckle tracking can detect subclinical functional abnormality even before any decrease in LV ejection fraction, we decided to conduct a study to evaluate the effect of bariatric surgery on cardiac function. Figure 1 shows the most common techniques of bariatric surgery.

The presenting study evaluated the effect of substantial weight loss early after bariatric surgery on cardiac function and myocardial deformation by 2D speckle echocardiography in morbidly obese patients early after bariatric surgery (at 3 and 6 months after surgery).

Multiple studies focused on the effects of bariatric surgery on the right ventricular size and function. They showed a favorable impact on RV size and function. However, prospective data on the short-term result of bariatric surgery on multiple guidelines recommended RV measurements still need to be included.

## Methods

### Study population

This was a prospective cohort study. Morbid obese patients scheduled for bariatric surgery from July 2017 to February 2018 at Firoozgar Hospital (Metabolic Surgery Center) were included in our study and referred for 2D speckle transthoracic echocardiography (TTE) in Rajaie Cardiovascular Medical and Research Center. The World Health Organization definition of morbid obesity (body mass index of ≥ 40 kg/m^2^ or ≥ 35 kg/m^2^ in patients with overweight-related comorbidity) was used in this study [12]. We excluded patients with uncontrolled hypertension, a history of cardiomyopathy, myocardial infarction, myocarditis, pericardial disease, moderate or severe valvular disease, any patient with wall motion abnormality, LVEF less than 50%, and technically poor image quality. Tachycardia and atrial fibrillation were among the other factors that were excluded. Forty-three consecutive patients were recruited in our study and underwent baseline TTE before surgery; Three patients were excluded due to suboptimal echocardiographic windows. Of the 40 patients with satisfactory echocardiographic views, 34 agreed to complete the study and underwent follow-up echocardiography 3 and 6 months after bariatric surgery.

### Echocardiographic evaluation

All examinations were performed with an EPIQ 7 (Philips) echocardiography machine using a Philips X5-1 xMATRIX array transducer for 2D echocardiography. Image acquisition was made according to echocardiography's recommendations for cardiac chamber quantification in adults [13].

Two-dimensional datasets were acquired with a sampling rate of 50–60 frames per second. At least two consecutive heartbeats were recorded for each image plane and stored digitally. Three apical views (apical four, three, and two chambers) for the measurement of global longitudinal strain (GLS) and three short axis views (at the level of the base, mid-papillary muscles, and apex) for the measurement of global circumferential strain (GCS) were stored. For offline strain analysis by QLAB software (version 10.8.5), aCMQ (Automated Cardiac Motion Quantification) was used. With aCMQ, the region of interest (ROI) was placed automatically based on the selected anatomical view. In suboptimal border detection, manual correction of endocardial borders was performed to generate measurements of global and regional myocardial functions. We excluded the patients if the endocardial border was not traceable in more than two segments in a view. Besides GLS and GCS, LV end-diastolic volume (EDV), end-systolic volume (ESV), and LV ejection fraction (EF) were automatically calculated using aCMQ software. LV mechanical dispersion index (MDI) is the standard deviation (SD) of the time-to-peak longitudinal and circumferential strain in each of the segments of the LV that were calculated by the software [14]. According to the chamber quantification guideline, the basal, mid, and long RV diameters were measured in an RV-focused view. In contrast, proximal RVOT was measured in the parasternal long-axis view [13].

**Fig. 2:**
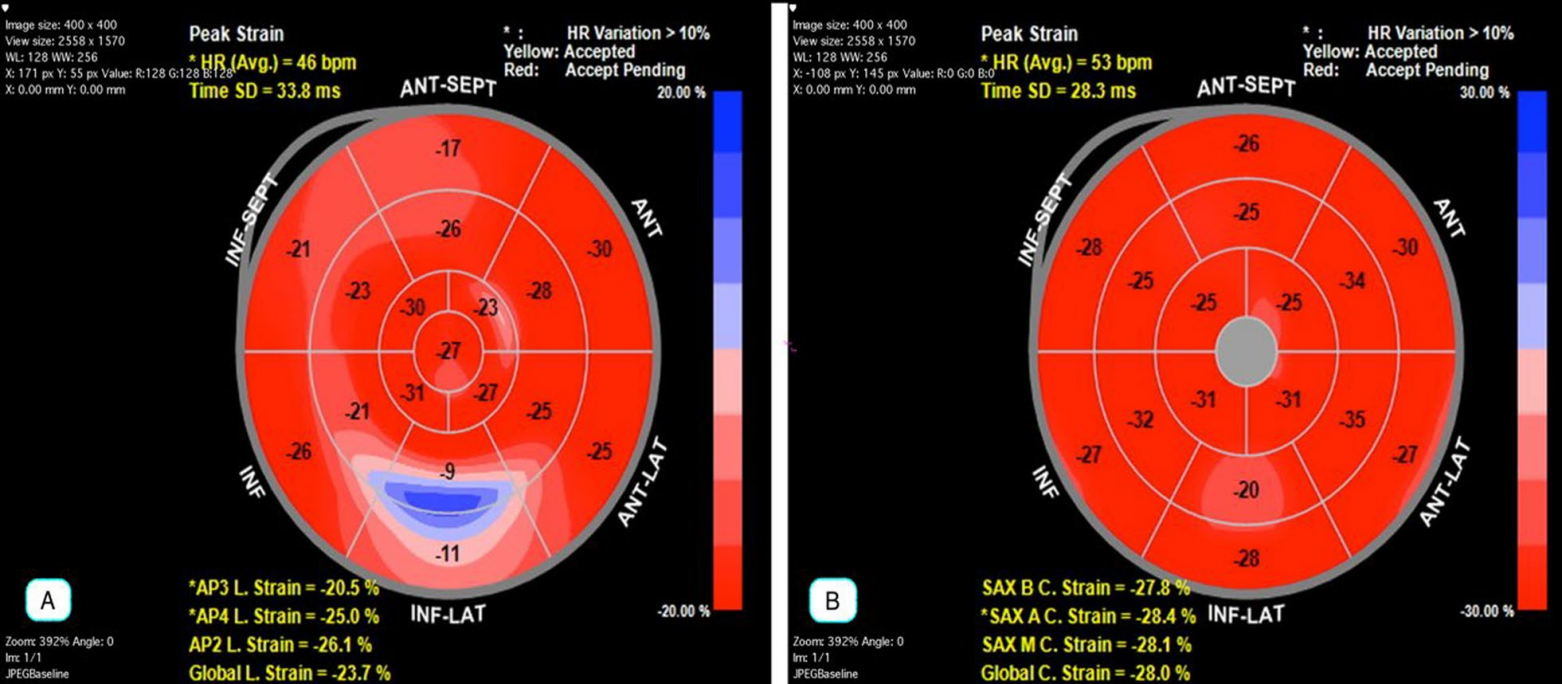
Bull’s eye diagram of the study population's global longitudinal strain (GLS) and global circumferential strain (GCS). **A** shows the Bull’s eye diagram of GLS and LV mechanical dispersion index of longitudinal strain. **B** shows the Bull’s eye diagram of GCS and LV mechanical dispersion index of circumferential strain

Figure 2 shows the Bull’s eye diagram of GLS and GCS and their mechanical dispersion index. Sugimoto's study analyzed the lower limit of normal LV strains with a vendor-independent software package and calculated them as ± 1.96 standard deviations from the mean. The minimum normal cutoff values for longitudinal strain were reported as − 16.7% in men and − 17.8% in women, and for circumferential strain, − 22.3% in men and − 23.6% in women [15].

### Study protocol

One person (echocardiographic fellowship) performed and analyzed all echocardiographic examinations. The strain study was repeated three and six months after surgery, and data were analyzed. The echocardiographer was blind to the amount of weight loss and type of bariatric surgery in each patient during strain analysis.

The study protocol was based on the Declaration of Helsinki and approved by the Ethics Committee of Rajaie Cardiovascular, Medical, and Research Center. Before enrollment, informed written consent was obtained from all patients.

### Data analysis

Finally, data were entered and analyzed using SPSS version 24 (SPSS Inc. Chicago Il, The USA). Results of quantitative variables are expressed as Mean ± SD and qualitative variables as no (%). The repeated-measures ANOVA test assessed the variable changes after 3 and 6 months. The post-HOC Bonferroni test was used in case of statistically significant differences. Comparing the abnormal right ventricular size percentage at different times was performed using Cochran's Q test. Cohen's d was used to assess the effect size. The association of weight loss with the change in GLS and GCS indices was analyzed with a simple linear regression test. The *P* value less than 0.05 was considered statistically significant.

## Results

### Baseline characteristics

Overall, 34 patients with complete data entered the study. The baseline characteristics of participants are shown in Table 1. Twenty-seven patients were female (79%), and seven were male (21%). The mean age of patients was 34.79 ± 8.4 years, with an age range of 20–55 years. Patients’ risk factors included cigarette smoking in 1(2%), diabetes in 3(6%), hypertension in 2(4%), dyslipidemia in 2(4%), and 28 patients (82%) who had no traditional risk factors. The body mass index (BMI) mean was 46.23 ± 5.35, with a range of 36.90–59.52 kg/m^2^. The types of bariatric surgery included 16 mini-bypass (47%), 10 sleeve (29.4%), and eight classic bypass surgeries (23.5%). Patients’ weight at baseline, 3, and 6 months after surgery was 130.14 ± 23.61, 106.50 ± 20.78, and 92.76 ± 18.93, respectively, with a statistically significant decrease (*P* value < 0.001) of weight in the first and second follow-ups. Mean weight loss at three months and six months was 23.64 ± 5.27 kg and 37.38 ± 8.77 kg, respectively.

**Table 1:** Baseline characteristics of the study participants

Variable	Value
Age, mean ± SD, years	34.79 ± 8.4
Gender	
Male, *n* (%)	7 (21%)
Female, *n* (%)	27 (79%)
Body mass index, mean ± SD, kg/m^2^	46.23 ± 5.35
Weight (kg)	
Baseline	130.14 ± 23.61
3 months	106.50 ± 20.78
6 months	92.76 ± 18.93
Type of surgery	
Mini bypass, *n* (%)	16 (47%)
Sleeve, *n* (%)	10 (29.4%)
Classic bypass, *n* (%)	8 (23.5%)

### 2D speckle echocardiography

The patients’ ejection fractions at baseline, 3, and 6 months after surgery were 58.45 ± 7.84%, 59.45 ± 6.93%, and 60.96 ± 6.45%, respectively, without a statistically significant difference.

**Fig. 3:**
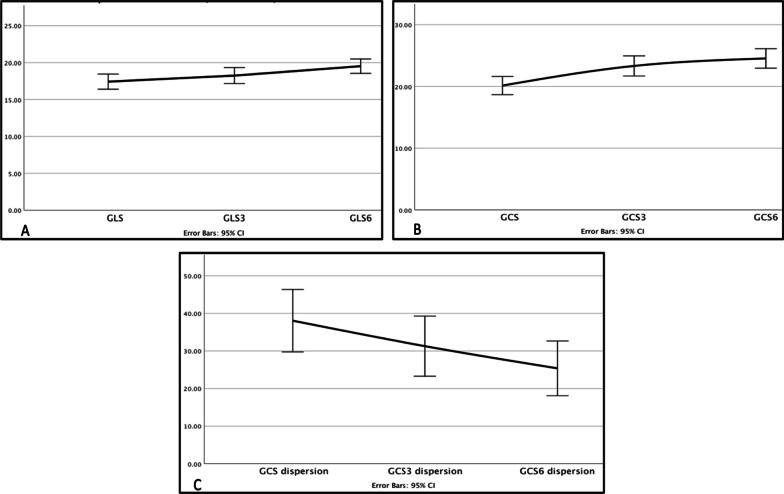
GLS, GCS, and mechanical dispersion index of circumferential strain changed during the follow-up compared to the baseline. Diagrams show the change in the absolute value of global longitudinal strain (**A**), global circumferential strain (**B**), and mechanical dispersion index of circumferential strain (**C**) before surgery compared to 3 and 6 months after the bariatric surgery

The absolute value of global longitudinal strain (GLS) at baseline, 3, and 6 months after surgery were 17.42 ± 2.94%, 18.24 ± 3.09%, and 19.52 ± 2.78%, respectively, with a statistically significant difference from baseline to six months after the surgery (*P* value < 0.001). The absolute value of global circumferential study (GCS) at baseline, 3, and 6 months after surgery was 20.14 ± 4.22%, 23.32% ± 4.66%, and 24.53 ± 4.52%, respectively, with statistically significant changes (*P* value < 0.001) from baseline to three months and from baseline to six months (0 → 3 and 0 → 6) and no significant difference from three months to six months after the surgery (no change in 3 → 6) (Fig. 3). This means that improvement in GCS occurred earlier than GLS in this study population.

Regarding the type of surgery, in the mini-bypass group, GCS was significantly lower than in classic and sleeve procedures (*P* value < 0.001).

A significant decrease was reported in mechanical dispersion of circumferential strain (38.05 ± 23.81–23.37 ± 20.86 ms, *P* value = 0.006) 6 months after surgery (Fig. 3).

### Correlation of change in GLS and GCS with the baseline values of GLS, GCS, weight loss, and baseline weight

**Fig. 4:**
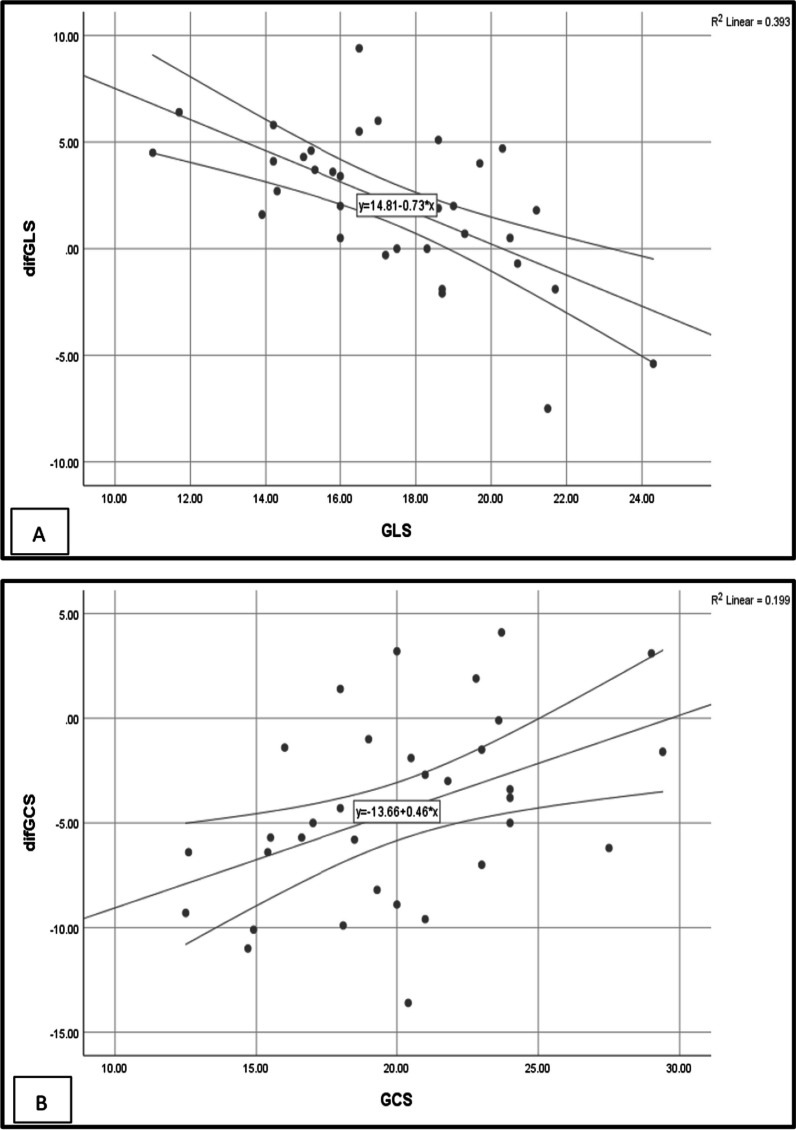
Improvement in GLS had a relatively strong negative correlation with the baseline GLS value (*R*^2^ = 0.39), while GCS changes had a weak positive correlation with baseline GCS (*R*.^2^ = 0.20)

The change in GLS and GCS after six months did not correlate with the baseline weight or weight loss (*P* value > 0.05), but they showed a correlation with the baseline GLS and GCS values (Fig. 4). As shown in Fig. 4a, GLS changes had a relatively strong negative correlation with the baseline GLS value (*R*^2^ = 0.39), while GCS changes had a weak positive correlation with baseline GCS (*R*^2^ = 0.20) (Fig. 4b).

### Assessment of right ventricular size

**Fig. 5:**
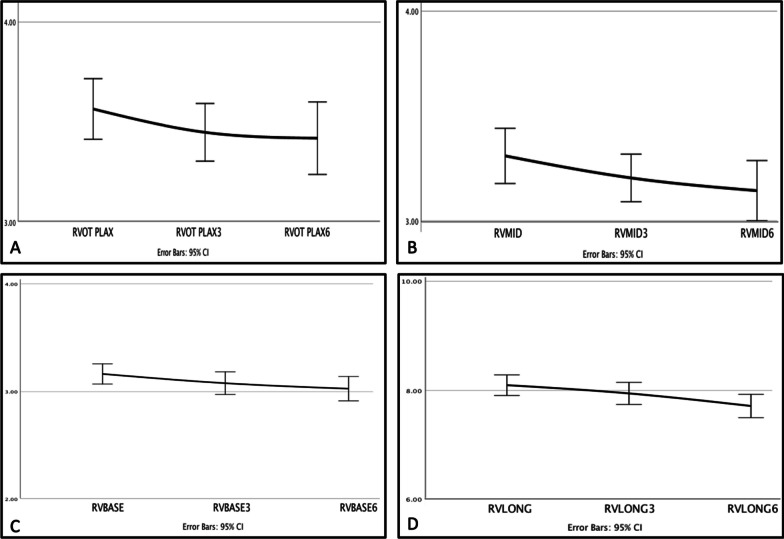
Changes in right ventricular size in different echocardiographic planes during the follow-up compared to baseline measurements. Mean RV diameter(centimeter) before surgery, at 3 and 6 months after surgery: proximal right ventricular outflow tract (RVOT) diameter in parasternal long-axis view (**A**), basal RV diameter (**B**), mid-RV diameter (**C**), and long-axis RV (**D**) in RV-focused view

Changes in other variables, including right ventricular (RV) size in multiple echocardiographic views, are presented in Table 2. Figure 5 shows a decreased RV diameter (RVD) in multiple echocardiographic planes. The right ventricular size in each echocardiographic plane was labeled as *“normal or abnormal”* based on the latest American Society Guideline 2016 [17] and is categorized in Table 3. The RV basal diameter of more than 41 mm, the RV mid-diameter of more than 35 mm, the RV longitudinal diameter of more than 83 mm, and the proximal right ventricular outflow (RVOT) diameter in the parasternal long axis (PLAX) view of more than 30 mm were considered abnormal. Before surgery, “basal RVD” was abnormal in none of the study populations, and “RVOT PLAX diameter” was abnormal in 91.2% of the patients, followed by the “long RVD” and “mid-RVD” that were abnormal (in 32.4% and 20.6% of the study group). A detailed workup of other causes of RV enlargement (including left to right shunts) was also carried out if indicated.

**Table 2:** A summary of study variable changes during the study period

Echocardiographic parameters	Baseline	3 months after the surgery	6 months after the surgery	Cohen’s *d* Baseline vs 6 months after surgery	*P* value
GLS (%)^a^	17.42 ± 2.94	18.24 ± 3.09	19.52 ± 2.79	0.73	0.001^*^ (0 → 6)
Longitudinal strain in AP4ch^b^ view (%)	18.41 ± 2.68	19.13 ± 2.54	20.98 ± 2.93	0.92	0.000^Ω^ (0 → 6 and 3 → 6)
Longitudinal strain in AP2ch^c^ view (%)	17.81 ± 3.21	18.75 ± 3.44	19.11 ± 2.72	0.44	0.053
Longitudinal strain in AP3ch^d^ view (%)	16.46 ± 3.89	17.86 ± 3.47	18.27 ± 3.40	0.50	0.021^*^ (0 → 6)
Mechanical dispersion of longitudinal strain (msec)	34.42 ± 17.84	30.55 ± 19.23	27.92 ± 23.08	− 0.32	0.117
GCS (%)^e^	20.14 ± 4.22	23.32 ± 4.66	24.53 ± 4.52	1.00	0.000^#^ (0 → 3 and 0 → 6)
Basal LV circumferential strain (%)	17.73 ± 4.52	20.19 ± 4.04	22.55 ± 5.51	0.96	0.000^Δ^ (0 → 3, 0 → 6 and 3 → 6)
Mid-LV circumferential strain (%)	20.11 ± 4.89	22.30 ± 5.74	24.41 ± 6.40	0.76	0.000^*^ (0 → 6)
Apical LV circumferential strain (%)	22.73 ± 6.15	26.71 ± 7.14	28.31 ± 7.85	0.79	0.000^#^ (0 → 3 and 0 → 6)
Mechanical dispersion of circumferential strain (msec)	38.05 ± 23.81	31.28 ± 22.92	25.37 ± 20.86	− 0.57	0.006^*^ (0 → 6)
RVOT PLAX (cm)^f^	3.56 ± 0.43	3.45 ± 0.41	3.42 ± 0.52	− 0.29	0.011^#^ (0 → 3 and 0 → 6)
Basal RVD (cm)^g^	3.16 ± 0.27	3.08 ± 0.30	3.03 ± 0.32	− 0.44	0.000^#^ (0 → 3 and 0 → 6)
Mid-RVD (cm)	3.31 ± 0.38	3.20 ± 0.32	3.14 ± 0.41	− 0.43	0.007* (0 → 6)
Long RVD (cm)	8.10 ± 0.54	7.95 ± 0.58	7.72 ± 0.61	− 0.66	0.001^Ω^ (0 → 6 and 3 → 6)
LVEF (%)^h^	58.45 ± 7.84	59.45 ± 6.93	60.96 ± 6.45	0.35	0.132
LVEDV (mL)^i^	103.7 ± 27.6	98.7 ± 28.9	95.4 ± 29.0	− 0.29	0.110
LVESV (mL)^j^	43.82 ± 16.63	40.70 ± 17.36	37.43 ± 14.19	− 0.41	0.037^*^ (0 → 6)

^a^GLS: global longitudinal strain; ^b^AP4ch: apical four-chamber view; ^c^AP2ch: apical two chamber; ^d^AP3ch: apical three chamber; ^e^GCS: global circumferential strain; ^f^RVOT PLAX: proximal right ventricular outflow tract diameter in parasternal view; ^g^RVD: right ventricular diameter; ^h^LVEF: left ventricular ejection fraction, ^i^LVEDV: left ventricular end-diastolic volume; ^j^LVESV: left ventricular end-systolic volume

^*^Statistically significant changes from baseline to six months after the surgery (0 → 6)

^#^Statistically significant changes from baseline to three months and six months (0 → 3 and 0 → 6) with no statistically significant change from three months to six months after the surgery (no change in 3 → 6)

^Ω^Statistically significant changes from baseline to six months and from three months to six months (0 → 6 and 3 → 6), with no statistically significant change from baseline to three months after the surgery (no change in 0 → 3)

^Δ^Statistically significant changes from baseline to three months, baseline to six months, and three months to six months after the surgery (0 → 3, 0 → 6 , and 3 → 6)

**Table 3:** Percentage of abnormal RV size* in different echocardiographic planes before surgery, three months, and six months after the bariatric surgery

Right ventricular size	Abnormal* (%)	Normal (%)	PV
RVOT PLAX^a^, baseline	91.2	8.8	0.121
RVOT PLAX, 3 months	82.4	17.6	
RVOT PLAX, 6 months	76.5	23.5	
Basal RVD^b^, baseline	0	100	1.000
Basal RVD, 3 months	0	100	
Basal RVD, 6 months	0	100	
Mid-RVD, baseline	20.6	79.4	0.565
Mid-RVD, 3 months	14.7	85.3	
Mid-RVD, 6 months	14.7	85.3	
Long RVD, baseline	32.4	67.6	0.178
Long RVD, 3 months	23.5	76.5	
Long RVD, 6 months	17.6	82.4	

^a^RVOT PLAX: right ventricular outflow tract diameter in parasternal view; ^b^RVD: right ventricular diameter

*RV size was considered abnormal based on the values provided in the latest “chamber quantification guideline” [17]. RV basal diameter > 41 mm, RV mid-diameter > 35 mm, RV longitudinal diameter > 83 mm, and proximal RVOT PLAX diameter > 30 mm are considered abnormal

Interestingly, RV diameters did not return to normal values six months after the surgery (Table 3). Although our study did not analyze systolic pulmonary artery pressure (sPAP), it was measured routinely in each patient before bariatric surgery. Only mild pulmonary hypertension was present in four patients (11.7%).

**Fig. 6:**
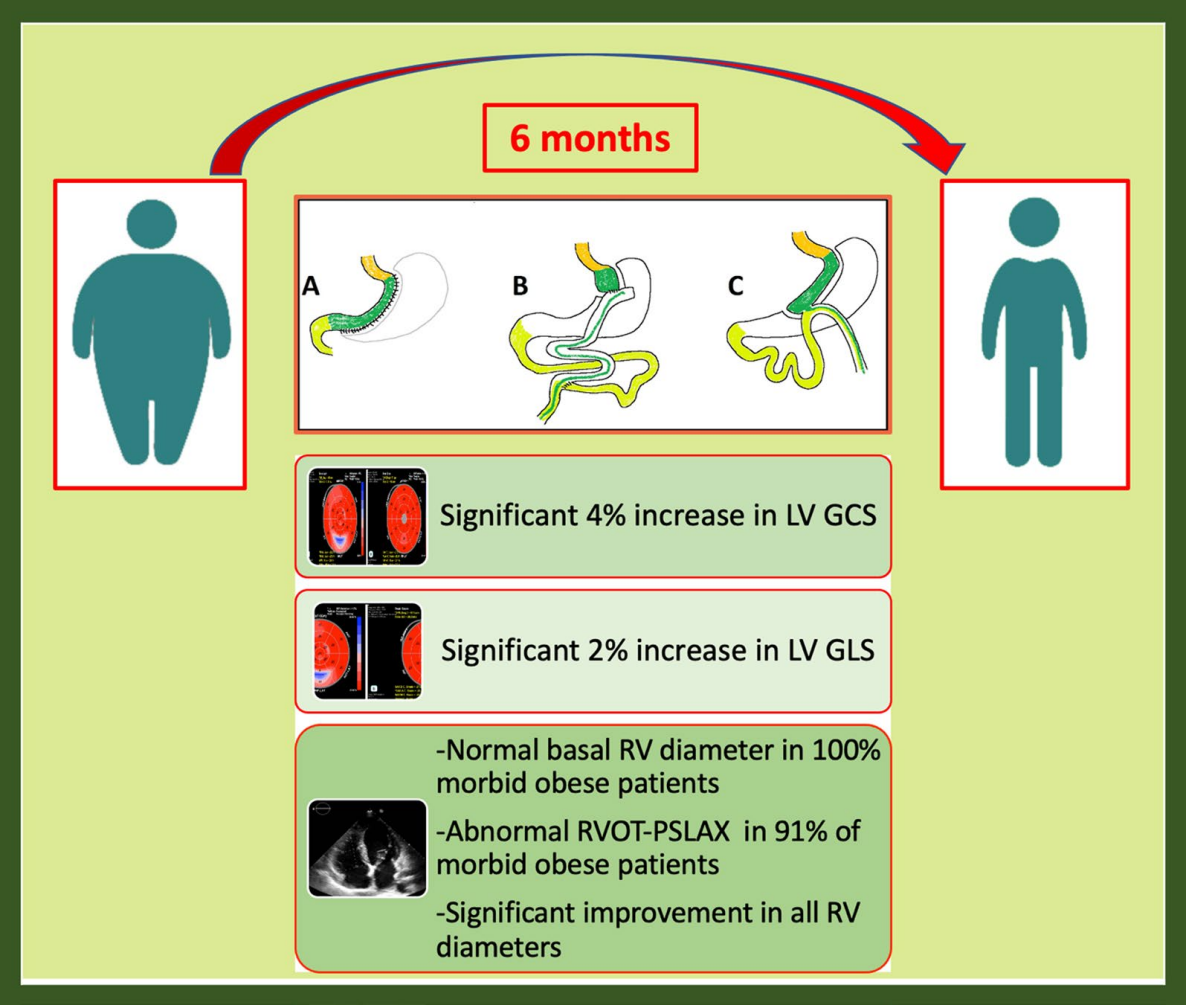
Bariatric surgery may improve GLS and GCS and affect right ventricular size after weight loss

A summary of the study findings was provided in the graphical abstract (Fig. 6).

## Discussion

Obesity remains one of the most important diseases of the twenty-first century, increasing the risk of heart failure, diabetes mellitus, dyslipidemia, sleep apnea, and cancers [16]. At first, it was thought that bariatric surgeries only restrict the intake volume; later research depicted the metabolic and systematic effects [17, 18]. It has been shown that morbid obesity can increase preload and afterload over time, leading to increased LV wall stress. LV dilatation and hypertrophy can ensue, increasing myocardial stiffness and resulting in LV diastolic and systolic dysfunction. Obesity is associated with activating the renin–angiotensin–aldosterone axis, which changes the cardiac structure and size [19, 20]. Expectedly, weight loss can reverse obesity-related cardiac changes if remodeling and fibrosis have not happened [21]. This supports the importance of early intervention, especially bariatric surgeries, at younger ages. Improved cardiac outcomes following bariatric surgery seem much further than decreased preload, afterload, and mechanical pressure on the myocardium [22].

Most studies generally assessed the favorable echocardiographic changes of bariatric surgery on the myocardium after six months. The presenting study tried to detect *early* echocardiographic changes in morbidly obese patients undergoing bariatric surgery. The main findings of this research were improved GLS, GCS, and dyssynchrony of circumferential movement of the left ventricle in six months after bariatric surgery. Despite significant weight loss three months after the surgery, only the LV's basal and apical circumferential strains showed improvement.

In a study by Grymyr in 2021, the one-year impact of bariatric surgery on LV mechanics was assessed, and the absolute value of GLS improvement was 4.6% at six months, and LVEF remained unchanged [23]. In multivariate regression analyses, 1-year improvement in GLS was predicted by lower preoperative GLS, more considerable mean blood pressure, and BMI reduction (all *P* < 0.05). In our study, the absolute increase in GLS was 2%, with no change in LVEF, and the improvement in GLS and GCS after six months did not necessarily correlate with the baseline weight or weight loss. As in Grymyr's study, the lower baseline GLS predicted more improvement in GLS six months after the surgery. Circumferential strains were not calculated in the Grymyr study.

In 2019, Santos et al. measured the GLS, GCS, GRS (global radial strain), and LV twist in 25 patients about three months after sleeve gastrectomy [24]. Only GLS improved by about 2%, and GCS, GRS, and LV twist remained unchanged. Contrarily, we found a significant increase in the absolute value of GCS (increment of 3% from baseline to three months after the surgery and 4% from baseline to six months after the surgery). Regarding the type of surgery, the absolute values of the GCS were significantly lower in the mini-bypass group than in classic surgery (at baseline and after three and six months) in our research. Unfortunately, our study sample size was not large enough to conclude the superiority of any procedure over the others.

A meta-analysis by Gherbesi, published in 2022, included 11 studies with follow-ups ≥ 6 months that confirmed GLS but not LVEF improved after bariatric surgery [25]. GCS was not an endpoint in this meta-analysis.

In another study in 2017 in South Korea, 37 patients undergoing bariatric surgery were studied by Shin et al. Echocardiography was performed before and after at least one year of bariatric surgery [26]. Bariatric surgery led to significant decreases in left ventricular (LV) size and mass and LV longitudinal strain (14.1 ± 1.9–16.2 ± 1.4%, *p* < 0.001 for longitudinal strain). Changes in LV longitudinal strain were related to LV mass reduction (*p* = 0.04). However, LV ejection fraction and LV circumferential and radial strains were comparable at follow-ups. Our study showed improved GLS, GCS, and dyssynchrony of circumferential movement of the left ventricle six months after bariatric surgery. Only the LV's basal and apical circumferential strains showed improvement three months after the surgery. GCS was not measured in Shin’s study.

In this study, the change in GLS and GCS after six months did not correlate with the baseline weight or weight loss, but it did correlate with the baseline GLS and GCS values. Further studies should reassess the mechanism behind different observed responses of longitudinal and circumferential strains to weight loss. The main practical finding was that patients with lower absolute GLS values had a greater increase in GLS after weight loss.

In this study, we did not assess blood pressure changes or metabolic alterations such as blood sugar level, as there was much evidence supporting their beneficial effects of improved metabolic state in morbidly obese patients after bariatric surgery. Enhanced glucose metabolism or the release of adipocytokines is responsible for these favorable outcomes [27, 28].

In practice, encountering an obese patient with RV enlargement usually requires multiple additional workups to exclude cardiac disorders, notably left-to-right shunts, and evidence of pulmonary hypertension. In contrast, the definite effect of obesity on RV size cannot be estimated. Unfortunately, the upper limit of normal RV size in obese patients has not been provided. Multiple studies focused on the effects of bariatric surgery on the right ventricular size and function; all showed a favorable impact on RV size and function [11]. In a retrospective cohort study, the changes in different RV sizes were assessed, and RV mid-cavity and longitudinal dimensions significantly decreased after surgery. They did not report the RV size as “normal” or” abnormal” based on the latest guidelines. The chamber quantification guideline [13] 2015 emphasized measuring RV size in an “RV-focused view” with suggestions to minimize the significant variability in acquiring RV views. The retrospective nature of their study (2008–2017) and the presence of different echocardiographers that may image RV before the widespread acceptance of the “RV-focused view” limits reliance on the change in RV size in the long follow after bariatric surgery [29].

We tried to elucidate the early effect of bariatric surgery on multiple echocardiographic right ventricular sizes and find which RV size shows the most remarkable change relative to the baseline measurement. In the presenting study, RVOT in PLAX view was abnormal in 91.2% of the study population at baseline and remained abnormal in 76.5% even after significant weight loss. On the other hand, basal RV diameter was normal in all of the participants. Despite the small study population, the following conclusions can be drawn from this study: First, the basal RV enlargement should not be explained by obesity, and other etiologies for RV enlargements, such as left to right shunt and pulmonary hypertension, should be in mind during echocardiography. Second, RVOT size in the PLAX view should not be relied on as a sole measurement for diagnosing RV enlargement in obese patients. Lastly, despite significant weight loss in six months after bariatric surgery, abnormal RV size did not return to the normal range in all participants. In a study by Eslami and colleagues [30], multiple echocardiographic planes were measured and indexed to body mass index (BMI) and body surface area (BSA) in 80 normal participants. They proposed a formula to predict maximum RV diameter based on BMI. The main problem with their proposed formula for estimating RV diameter in obese patients was the small number of people with a BMI of more than 25 kg/m^2^ in this study. Long-term studies of the effect of bariatric surgery on the right ventricular size are needed to find the threshold of abnormal RV diameter in obese patients.

### Limitations

Detailed clinical and laboratory data of the patients were documented in the registry of metabolic surgery at Firoozgar Hospital, and patients were referred to us only if they met the criteria. As we aimed to assess the early cardiac effect of weight loss, patients with clinical conditions that may have concurrent cardiac effects, such as uncontrolled hypertension, a history of cardiomyopathy, myocardial infarction, myocarditis, pericardial disease, and moderate or severe valvular disease, were excluded. Tachycardia and atrial fibrillation were among the other risk factors that were excluded. Laboratory data, including kidney function, lipids, and pro-BNP, were documented in the Firoozgar database but were not included in our research as we solely focused on echocardiographic changes.

Bariatric surgery and 2D speckle echocardiography were performed in two different hospitals, which resulted in the loss of follow-up. After significant weight loss, some patients did not return for echocardiographic follow-up. Endocardial detection and performing strain studies are challenging in morbidly obese patients. We tried to overcome this limitation by including patients with satisfactory views, and obese patients were excluded if the endocardial border was not traceable in more than two segments in a view. As we excluded patients with underlying cardiac disease (cardiomyopathies, history of previous myocardial infarction or myocarditis), generalization of the study results to these groups of patients is impossible. The reason behind this strict exclusion criteria was to attribute the observed influence to the direct effect of weight loss following bariatric surgery. Clarifying why GCS improvement precedes GLS and its explanation requires further investigation with a larger sample size to elucidate the early impact of bariatric surgery on enhancing layer-specific strain. This study assessed only proximal RVOT in the parasternal long-axis view, as acquiring the true short-axis view of the RVOT needs stable and constant landmarks, which is challenging in obese patients.

Besides, as we did not evaluate patients’ clinical outcomes, taking this improvement in GLS and GCS and mechanical dispersion into clinical practice is not applicable. Considering RV size in echocardiography is challenging, and adhering to chamber quantification guidelines was pursued to minimize the variability in RV measurement. Due to the small sample size, defining a new threshold for abnormal RV size was impossible. The lack of significant changes in RV size post-surgery despite significant weight loss may be due to the early time of follow-ups and the relatively healthy groups of morbidly obese patients. Again, the small study population prevents extrapolating the improvement of 2D speckle indices and RV size to all post-bariatric surgery patients.

### Conclusions

Speckle-tracking echocardiography has proven that bariatric surgery may enhance left ventricular function. Changes in right ventricular size should be considered and assessed during echocardiography in obese patients after weight loss.

## Data Availability

The study's data are available for further analysis upon the reasonable request of the corresponding author.

## Abbreviations

LV: Left ventricular
LVH: Left ventricular hypertrophy
HF: Heart failure
RV: Right ventricular
TTE: Transthoracic echocardiography
GLS: Global longitudinal strain
GCS: Global circumferential study
SDSTE: 2D speckle tracking echocardiography
EF: LV ejection fraction
ESV: End-systolic volume
EDV: End-diastolic volume
MDI: LV mechanical dispersion index
SD: Standard deviation
BMI: Body mass index
RVD: RV diameter
RVOT: Right ventricular outflow
PLAX: Parasternal long axis

## References

[1] de Witte D, Wijngaarden LH, van Houten VA, van den Dorpel MA, Bruning TA, van der Harst E (2020). Improvement of cardiac function after Roux-en-Y gastric bypass in morbidly obese patients without cardiac history measured by cardiac MRI. Obes Surg.

[2] Giudici A, Palombo C, Kozakova M, Morizzo C, Losso L, Nannipieri M (2020). Weight loss after bariatric surgery significantly improves carotid and cardiac function in apparently healthy people with morbid obesity. Obes Surg.

[3] Park J-B, Kim DH, Lee H, Hwang I-C, Yoon YE, Park HE (2020). Obesity and metabolic health status are determinants for the clinical expression of hypertrophic cardiomyopathy. Eur J Prev Cardiol.

[4] Cooiman M, Kleinendorst L, Aarts E, Janssen I, van Amstel HP, Blakemore AI (2020). Genetic obesity and bariatric surgery outcome in 1014 patients with morbid obesity. Obes Surg.

[5] English WJ, DeMaria EJ, Hutter MM, Kothari SN, Mattar SG, Brethauer SA (2020). American Society for Metabolic and Bariatric Surgery 2018 estimate of metabolic and bariatric procedures performed in the United States. Surg Obes Relat Dis.

[6] Mikhail N, Golub MS, Tuck ML (1999). Obesity and hypertension. Prog Cardiovasc Dis.

[7] Rahmouni K, Correia ML, Haynes WG, Mark AL (2005). Obesity-associated hypertension: new insights into mechanisms. Hypertension.

[8] Choromańska B, Myśliwiec P, Łuba M, Wojskowicz P, Myśliwiec H, Choromańska K (2020). The impact of hypertension and metabolic syndrome on nitrosative stress and glutathione metabolism in patients with morbid obesity. Oxid Med Cell Longev.

[9] Choromańska B, Myśliwiec P, Łuba M, Wojskowicz P, Myśliwiec H, Choromańska K (2020). Impact of weight loss on the total antioxidant/oxidant potential in patients with morbid obesity—a longitudinal study. Antioxidants.

[10] Dwivedi AK, Dubey P, Cistola DP, Reddy SY (2020). Association between obesity and cardiovascular outcomes: updated evidence from meta-analysis studies. Curr Cardiol Rep.

[11] Cuspidi C, Rescaldani M, Sala C, Grassi G (2014). Left-ventricular hypertrophy and obesity: a systematic review and meta-analysis of echocardiographic studies. J Hypertens.

[12] Sümer A (2016). Definitions of obesity and current indications for obesity surgery. Laparosc Endosc Surg Sci (LESS).

[13] Lang RM, Badano LP, Mor-Avi V, Afilalo J, Armstrong A, Ernande L (2015). Recommendations for cardiac chamber quantification by echocardiography in adults: an update from the American Society of Echocardiography and the European Association of Cardiovascular Imaging. Eur H J Cardiovasc Imaging.

[14] Mele D, Trevisan F, Fiorencis A, Smarrazzo V, Bertini M, Ferrari R (2020). Current role of echocardiography in cardiac resynchronization therapy: from cardiac mechanics to flow dynamics analysis. Curr Heart Fail Rep.

[15] Sugimoto T, Dulgheru R, Bernard A, Ilardi F, Contu L, Addetia K, Caballero L, Akhaladze N, Athanassopoulos GD, Barone D, Baroni M (2017). Echocardiographic reference ranges for normal left ventricular 2D strain: results from the EACVI NORRE study. Eur Heart J Cardiovasc Imaging.

[16] Sturm R (2007). Increases in morbid obesity in the USA: 2000–2005. Public Health.

[17] Kashyap SR, Bhatt DL, Wolski K, Watanabe RM, Abdul-Ghani M, Abood B (2013). Metabolic effects of bariatric surgery in patients with moderate obesity and type 2 diabetes: analysis of a randomized control trial comparing surgery with intensive medical treatment. Diabetes Care.

[18] Sinclair P, Docherty N, le Roux CW (2018). Metabolic effects of bariatric surgery. Clin Chem.

[19] Lambert EA, Straznicky NE, Dixon JB, Lambert GW (2015). Should the sympathetic nervous system be a target to improve cardiometabolic risk in obesity?. Am J Physiol Heart Circ Physiol.

[20] Cooper SA, Whaley-Connell A, Habibi J, Wei Y, Lastra G, Manrique C (2007). Renin-angiotensin-aldosterone system and oxidative stress in cardiovascular insulin resistance. Am J Physiol Heart Circ Physiol.

[21] Lavie CJ, Milani RV, Ventura HO (2009). Obesity and cardiovascular disease: risk factor, paradox, and impact of weight loss. J Am Coll Cardiol.

[22] Mandviwala T, Khalid U, Deswal A (2016). Obesity and cardiovascular disease: a risk factor or a risk marker?. Curr Atheroscler Rep.

[23] Grymyr LMD, Nadirpour S, Gerdts E, Nedrebø BG, Hjertaas JJ, Matre K, Cramariuc D (2021). One-year impact of bariatric surgery on left ventricular mechanics: results from the prospective FatWest study. Eur Heart Jo Open.

[24] Santos ECL, del Castillo JM, Parente GBO, Pedrosa RP, Gadelha PS, Lopes RD (2020). Changes in left ventricular mechanics after sleeve gastrectomy. Obes Surg.

[25] Gherbesi E, Cuspidi C, Faggiano A, Sala C, Carugo S, Tadic M (2022). Bariatric surgery and myocardial mechanics: a meta-analysis of speckle tracking echocardiographic studies. J Clin Med.

[26] Shin S-H, Lee YJ, Heo Y-S, Park S-D, Kwon S-W, Woo S-I (2017). Beneficial effects of bariatric surgery on cardiac structure and function in obesity. Obes Surg.

[27] Perego L, Pizzocri P, Corradi D, Maisano F, Paganelli M, Fiorina P (2005). Circulating leptin correlates with left ventricular mass in morbid (grade III) obesity before and after weight loss induced by bariatric surgery: a potential role for leptin in mediating human left ventricular hypertrophy. J Clin Endocrinol Metab.

[28] Mukerji R, Petruc M, Fresen JL, Terry BE, Govindarajan G, Alpert MA (2012). Effect of weight loss after bariatric surgery on left ventricular mass and ventricular repolarization in normotensive morbidly obese patients. Am J Cardiol.

[29] Sorimachi H, Obokata M, Omote K, Reddy YN, Takahashi N, Koepp KE, Ng AC, Rider OJ, Borlaug BA (2022). Long-term changes in cardiac structure and function following bariatric surgery. J Am Coll Cardiol.

[30] Eslami M, Larti F, Larry M, Molaee P, Badkoobeh RS, Tavoosi A, Safari S, Parsa AF (2017). Two-dimensional echocardiographic right ventricle measurements adjusted to body mass index and surface area in a normal population. J Clin Ultrasound.

